# Evaluation of Mutagenicity and Anti-Mutagenicity of Various Bean Milks Using *Drosophila* with High Bioactivation

**DOI:** 10.3390/foods11193090

**Published:** 2022-10-05

**Authors:** Woorawee Inthachat, Uthaiwan Suttisansanee, Kalyarat Kruawan, Nattira On-Nom, Chaowanee Chupeerach, Piya Temviriyanukul

**Affiliations:** Food and Nutrition Academic and Research Cluster, Institute of Nutrition, Mahidol University, Salaya, Phuttamonthon, Nakhon Pathom 73170, Thailand

**Keywords:** anti-mutagenicity, beans, *Drosophila*, Fabaceae, genotoxicity, mutagenicity, risk assessment, wing spot test

## Abstract

The consumption of a nutritious diet including phytochemicals can minimize mutations as the primary cause of carcinogenesis. Bean consumption supplies calories, minerals and phytochemicals but their anti-mutagenic properties in vivo remain little understood. Hence, the present study aimed to study the mutagenicity and anti-mutagenic properties of five bean milks using the somatic mutation and recombination test (SMART) involving *Drosophila* with high bioactivation. Milk derived from five bean varieties, namely black bean (*Phaseolus vulgaris*), red kidney bean (*Phaseolus vulgaris*), mung bean (*Phaseolus aureus*), peanut (*Arachis hypogaea*) and soybean (*Glycine max*) did not induce DNA mutations in *Drosophila* with high bioactivation, indicating their genome-safe properties. All bean milks showed anti-mutagenicity against the food-derived mutagen, urethane, in vivo with different degrees of inhibition. In the co-administration study, larvae were treated with each bean milk together with urethane. Soybean milk showed the highest anti-mutagenicity at 27.75%; peanut milk exhibited the lowest at 7.51%. In the pre-feeding study, the larvae received each bean milk followed by urethane. Soybean milk exhibited the highest anti-mutagenic potential, followed by red kidney bean and black bean milks. Total phenolic and antioxidant data revealed that the anti-mutagenicity of both red kidney bean milk and black bean milk might be derived from their phenolic or antioxidant properties; other phytochemicals may contribute to the high anti-mutagenicity observed in soybean milk. Further investigations on the anti-mutagenicity of bean milks against other dietary mutagens are required to develop bean-based products with potent anti-mutagenic properties.

## 1. Introduction

A mutation is a change in DNA sequences or chromosomal architecture influenced by exposure to chemicals known as mutagens. There are two types of mutations—germline mutations and somatic mutations. Germline mutations occur in eggs and sperm and can be passed on to offspring; somatic mutations occur solely in body cells [1]. Mutations have been well-documented as an underlying cause of carcinogenesis, particularly in genes implicated in apoptosis and proliferation [2], resulting in uncontrolled cell division and death [3]. Interestingly, 90–95% of cancers are caused by somatic mutations; only 5–10% are inherited as germline mutations [4]. The International Agency for Research on Cancer (IARC) reported that cancer was the leading cause of death worldwide in 2020 for more than 10 million people. The most common cancers are lung cancer, colon cancer and liver cancer [5]. In 2019, the National Cancer Institute reported the highest cancer cases in Thailand as liver and bile duct cancers at 19.5% in males; 40% of females were diagnosed with breast cancer [6]. Therefore, adopting a healthy lifestyle and avoiding exposure to mutagens such as mycotoxins, heavy metals, food-derived genotoxins, free radicals and even sunlight may help to prevent cancer in an estimated 30–50% of cases [7]. Furthermore, the consumption of healthy foods may also ameliorate cancer occurrence as some foods have cancer-fighting properties. For example, ingestion of at least three servings of citrus fruit per week reduced stomach cancer risk by 28% [8]. Consuming fruit and vegetables may help to prevent cancer development because these foods possess numerous phytochemicals that exhibit health benefits including anti-inflammatory, anti-cancer and antioxidant properties [9]. A multisite case-control study in Uruguay covering 3539 cancer cases and 2032 hospital controls revealed that reduced cancer risk of the upper aerodigestive tract, stomach, colorectum and kidney was affiliated with legume consumption [10]. Consumption of beans and legumes may protect against colon cancer because they are rich in phytochemicals as well as fiber. A study by the European Prospective Investigation into Cancer and Nutrition (EPIC) with 11 years of follow-up recommended increasing the consumption of fiber for colorectal cancer prevention [11].

Beans, which are seeds of plants in the family Fabaceae (or Leguminosae) are cultivated and consumed throughout the world as an essential nutritional and economic human and animal food crop. Beans are nutrient-rich and contain protein, carbohydrate, resistant starch, dietary fiber, potassium, calcium and folate (vitamin B9) [12,13]. Beans are also sources of phenolic acids and flavonoids including isoflavones, saponins and tannins [14,15]. The black bean seed coat of the Negro San Luis variety contains total flavonoids at 765.50 mg/100 g of sample, with significant contents of quercetin 4-*O*-galactoside and myricetin 3-*O*-glucoside [15]; UI 911 black beans exhibit three major anthocyanins as delphinidin 3-*O*-glucoside (56%), petunidin 3-*O*-glucoside (26%) and malvidin 3-*O*-glucoside (18%) [16], and mung bean (*Vigna radiata*) contains sinapic acid, *p*-coumaric acid and *t*-ferulic acid as primary phenolic acids [17]. Phytochemicals have recently received considerable attention because of their health-promoting properties including anti-cancer, antioxidant and anti-mutagenic activities. Interestingly, antioxidant properties have also been proposed to prevent mutations [18]. Thus, beans may exhibit anti-mutagenicity and play a preventive role against mutation-related disorders. To prove this hypothesis, this study evaluated the antioxidant, mutagenic and anti-mutagenic properties against a standard food-derived mutagen of five beans commonly consumed in Thailand, namely black bean (*Phaseolus vulgaris* L.), mung bean (*Vigna radiata* (L.) R. Wilczek), peanut (*Arachis hypogaea* L.), red kidney bean (*Phaseolus vulgaris* L.) and soybean (*Glycine max* (L.) Merr.). The somatic mutation and recombination test (SMART) or wing spot assay that detects the in vivo genotoxicity of chemicals using *Drosophila melanogaster* was employed as a model [19]. Several current studies take advantage of the SMART assay, including its high sensitivity to detect wide ranges of mutations and chromosomal aberrations [20,21]. Moreover, the *Drosophila* model possesses several advantages over other in vitro model organisms such as covering more diverse tests than bacteria and being cheaper than rodents. The fly strains used in this study exhibited high Phase I enzyme expression, cytochrome P450, which is a major enzyme contributing to the xenobiotic biotransformation in mammals, therefore allowing detection of both direct and indirect-acting mutagens. Information gained from this study will promote bean-based functional food development with anti-mutagenic properties.

## 2. Materials and Methods

### 2.1. Chemicals

Methanol (CH_3_OH, CAS No. 67-56-1, 99.9% purity), 2,3,5-triphenyltetrazolium chloride or TPTZ (C_19_H_15_C_l_N_4_, CAS No. 298-96-4, 98% purity), hydrochloric acid (HCl, CAS No. 7647-01-0), ferric chloride (FeCl_3_, CAS No. 7705-08-0, 97% purity), ferrous sulfate (FeSO_4_, CAS No. 7782-63-0, 99% purity) and Folin–Ciocalteu reagent were purchased from Sigma-Aldrich (St. Louis, MO, USA). Gallic acid (CAS No. 5995-86-8, 98% purity) and urethane or ethyl carbamate (CAS No. 51-79-6, 98% purity) were purchased from Tokyo Chemical Industry (Tokyo, Japan).

### 2.2. Sample Preparation

Five beans, namely black bean (*Phaseolus vulgaris* L.), mung bean (*Vigna radiata* (L.) R. Wilczek), peanut (*Arachis hypogaea* L.), red kidney bean (*Phaseolus vulgaris* L.), and soybean (*Glycine max* (L.) Merr.) were collected from convenience stores in Bangkok, Thailand. Plant names were rechecked with http://www.theplantlist.org, accessed on 9 April 2022.

For bean milk SMART assay preparation, a 200 g sample of each bean was washed in water and soaked for 12 h at room temperature. After soaking, the water was drained, and the beans were rinsed twice with drinking water. The sample was blended with 1.8 L of drinking water and filtered through a lawn sieve. Thus, water: bean seed = 9:1, *v/w*) as previously described [22]. The aqueous solution was boiled at 95–100 °C for 30 min, and the obtained bean milk was filtered and stored at −20 °C until required for use.

For antioxidant and total phenolic analyses, 50 g of each bean sample were cleaned twice with distilled water to remove debris and then extracted by boiling with 150 mL of 95–100 °C distilled water for 15 min. One gram of boiled bean was meshed and placed in a test tube containing 40 mL of methanol/water (50:50, *v/v*). The tube was thoroughly shaken at room temperature for 1 h, then centrifuged at 2500× *g* for 10 min and the supernatant was recovered, filtered and stored at −20 °C until required for analysis.

### 2.3. Somatic Mutation and Recombination Test (SMART) or Wing Spot Test

Two strains of *Drosophila* were used, namely (i) the *mwh* strain (with genetic constitution *y; mwh j*), containing wing cell marker *multiple wing hair (mwh)* and (ii) the ORR strain (with genetic constitution ORR; *flr^3^/In (3LR) TM3, ri p^p^ sep l(3)89Aa bx^34e^ eBd^s^*), containing chromosomes 1 and 2 from DDT-resistant Oregon R(R) line. Thus, crossing these two strains resulted in F1 progenies expressing cytochrome P450 enzymes involved in xenobiotic biotransformation [23]. The insects were maintained at 25 ± 1 °C and 60–70% RH at photoperiod 12:12 (light:dark) on yeast–glucose–agar *Drosophila* medium (regular medium). In brief, corn flour (2.50 g), sugar (2 g), agar (0.28 g) and yeast (1 g) were mixed and boiled in a 50 mL beaker containing 20 mL deionized water (DI) until it became sticky. Propionic acid (0.10 mL) was added to the medium as a preservative. During food preparation, DI was replaced with bean milk in the experimental medium resulting in the final concentration of each bean milk at 111 mg/mL.

#### 2.3.1. Mutagenic Evaluation of Bean Milks Using the Co-Administration Study

**Figure 1:**
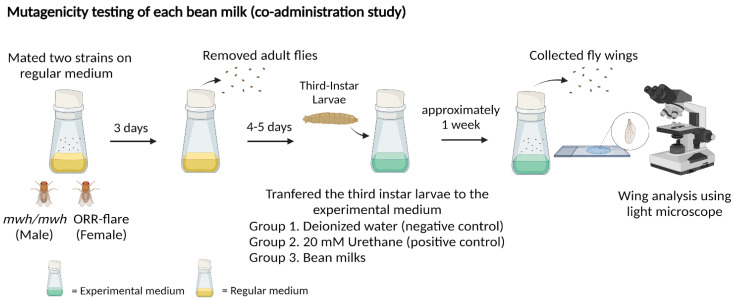
Experimental design of the co-administration study for mutagenicity testing: crossed adult flies were fed on the regular medium and the third instar larvae were transferred to the experimental medium containing each bean milk, water (negative control) or 20 mM urethane (positive control). Wings were collected and analyzed for mutant spots.

The *mwh* strain and the ORR strain were bred on the regular medium to produce trans-heterozygous larvae (F1 progenies). About 6 d after mating, one hundred of 3d old larvae were collected and transferred to (i) regular medium without bean milk or (ii) regular medium containing bean milk or urethane (experimental medium). DI was used as a negative control and 20 mM urethane was used as a mutagen. The larvae were fed on each medium until pupation at 25 °C. After metamorphosis, the surviving adult flies were collected, and the wings were removed for mutant spot analysis. Approximately 40 wings were scored for each medium. Results were expressed as the frequencies of small, large and twin spots. The experimental flow chart for the co-administration study is depicted in Figure 1.

#### 2.3.2. Mutagenic Evaluation of Bean Milks Using the Pre-Feeding Study

**Figure 2:**
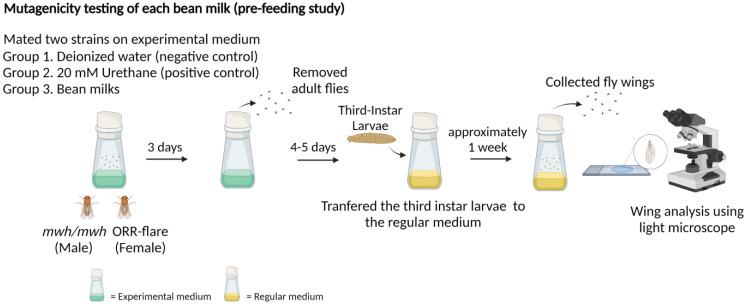
Experimental design of the pre-feeding study for mutagenicity testing: crossed adult flies were fed on the experimental medium containing each bean milk, water (negative control) or 20 mM urethane (positive control), and the third instar larvae were transferred to the regular medium. Wings were collected and analyzed for mutant spots.

The *mwh* strain and the ORR strain were bred on the experimental medium containing bean milk or urethane to produce F1 progenies. The larvae were then transferred to a fresh experimental medium and cultured at 25 °C until pupation. After metamorphosis, the surviving flies were collected and the wings were removed for mutant spot analysis. The experimental design for the pre-feeding study is illustrated in Figure 2.

#### 2.3.3. Anti-Mutagenic Evaluation of Bean Extracts Using the Co-Administration Study

**Figure 3:**
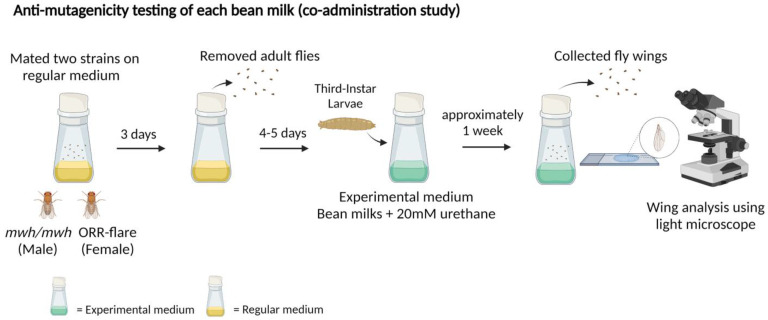
Experimental design of the co-administration study for anti-mutagenicity testing: crossed adult flies were fed on the regular medium, and the third instar larvae were transferred to the experimental medium containing each bean milk + 20 mM urethane. Wings were collected and analyzed for mutant spots.

The *mwh* strain and the ORR strain were bred on the regular medium to produce trans-heterozygous larvae (F1 progenies). After that, one hundred of 3d old larvae were collected and transferred to (i) regular medium containing only urethane and (ii) regular medium containing each bean milk + urethane. The larvae were fed on each medium until pupation at 25 °C. After hatching, the surviving adult flies were collected, and the wings were removed for mutant spot analysis. The experimental flow chart is depicted in Figure 3. Wing mutation values were also calculated for anti-mutagenicity based on a previous study [24,25]. Based on the control-corrected spot frequencies, percentage of anti-mutagenicity of each bean extract was calculated as in Equation (1).

(1) $$\%\;\mathrm{Anti}-\mathrm{mutagenicity}=(1-(\frac{b}{a}))\times 100$$

where *a* is the number of spots per wing induced by urethane, and *b* is the number of spots per wing induced by urethane in the presence of each bean extract.

#### 2.3.4. Anti-Mutagenic Evaluation of Bean Milks Using the Type I Pre-Feeding Study

**Figure 4:**
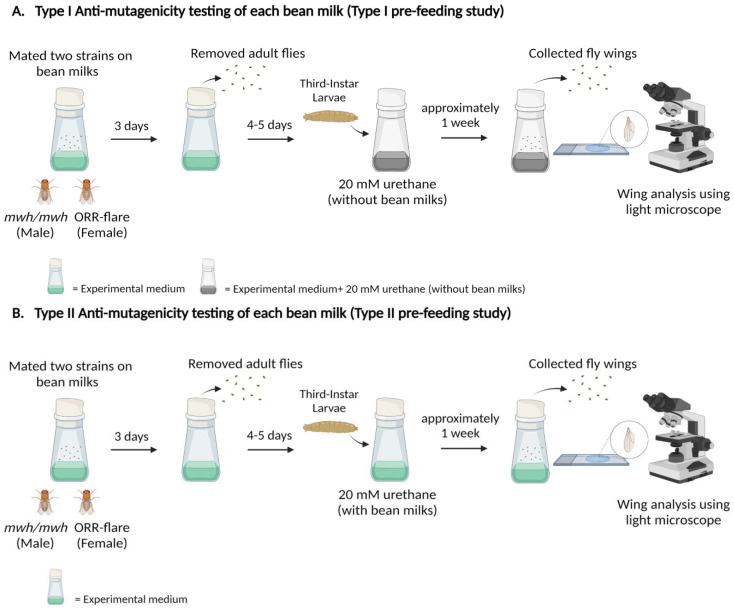
Experimental design of the pre-feeding study for anti-mutagenicity testing: (**A**) Pre-feeding study type I; crossed adult flies were fed on the experimental medium containing only bean milk, and the third instar larvae were transferred to the experimental medium containing only 20 mM urethane. Wings were collected and analyzed for mutant spots, (**B**) Pre-feeding study type II; crossed adult flies were fed on the experimental medium containing only bean milk, and the third instar larvae were transferred to the experimental medium containing each bean milk + 20 mM urethane. Wings were collected and analyzed for mutant spots.

The *mwh* and ORR strains were bred on the experimental medium containing bean milk to produce F1 progenies. After that, one hundred of 3d old larvae were transferred to the regular medium containing only urethane. After hatching, the wings were collected for mutant spot analysis. The experimental procedure is shown in Figure 4A.

#### 2.3.5. Anti-Mutagenic Evaluation of Bean Milks Using the Type II Pre-Feeding Study

The *mwh* and ORR strains were bred on the experimental medium containing bean milk to produce F1 progenies. After that, one hundred of 3d old larvae were transferred to the experimental medium containing bean milk + urethane. After hatching, the wings were collected for mutant spot analysis. The experimental procedure is shown in Figure 4B.

### 2.4. Ferric Reducing Antioxidant Power (FRAP) Assay

The ferric reducing antioxidant power (FRAP) assay was measured as described by Griffin and Bhagooli [26]. In a 96-wells plate, 150 µL of FRAP reagent (was prepared by mixing 300 mM acetate buffer, 10 mL TPTZ in 40 mM HCl and 20 mM FeCl_3_.6H_2_O in the proportion of 10:1:1 at 37 °C). A blank reading was then taken at 600 nm using a microplate plate reader. A 20 µL of sample extract or ferrous sulfate (FeSO_4_) standard (concentration 62.5, 125, 250, 500, 1000 µM) were then added to each well. The change in absorbance from the initial blank was read after 8 min. Absorbance was read at 600 nm. Results were expressed as mM FeSO_4_ equivalent per gram of dry weight (DW).

### 2.5. Total Phenolic Content Assay

The total phenolic content assay was measured as described by Amarowicz et al. [27], and modified the procedures of measurement using a microplate reader. A 10 µL of Folin–Ciocalteu reagent, 10 µL sample extract, standard (gallic acid) or blank, and 160 µL of distilled water, were added to a 96-well plate; this mixture was allowed to stand for 5 min before the addition of 20 µL of a saturated sodium carbonate solution. The plate was mixed well, and the absorbance of blue mixtures was recorded at 750 nm with microplate reader after 30 min incubation. The readings of sample and reagent blanks were subtracted from the reading of reagent with extract. The total phenolic contents (TPCs) were calculated as a gallic acid equivalent (GAE) from a calibration curve of gallic acid standard solutions (ranging from 25 to 800 mM) and expressed as mg GAE per gram of DW.

### 2.6. Statistical Analysis

The significances of mutant spots compared to negative or positive control were calculated as a previously described by Frei & Wurgler [28]. For survival assay, antioxidant activities and TPCs, the one-way analysis of variance (ANOVA) with indicated post hoc were used to state significant difference among values. Statistically analyses were performed using the statistical package for the social sciences (version 18 for Windows, SPSS Inc., Chicago, IL, USA). The principal component analysis (PCA) was analyzed using XLSTAT^®®^ (Addinsoft Inc., New York, NY, USA).

## 3. Results

### 3.1. Mutagenic Evaluation of Bean Milks Using the Co-Administration Study

**Figure 5:**
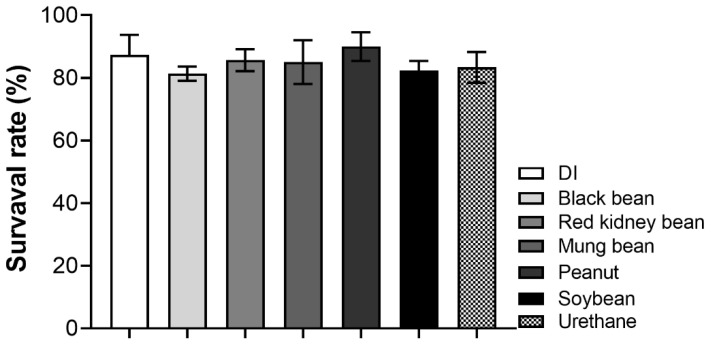
The percentage of *Drosophila* survival rate cultured on DI, 20 mM urethane or bean milk. Compared to the control group, the survival rate of larvae exposed to each bean milk was not statistically significant calculated by one-way analysis of variance (ANOVA) and Tukey’s multiple comparison test. The data were collected from three independent experiments (*n* = 3) and are expressed as mean ± SD.

To ascertain the non-toxic effects of each bean milk, a survival assay was performed. In brief, one hundred F1 larvae were fed on the regular medium containing bean milk, water (DI) or urethane. Within 7 d after hatching, the number of flies was counted and compared with DI and the mutagen (urethane). Results in Figure 5 show that all bean milks exhibited survival rates ranging from 80 to 90% and were comparable with the negative control (DI) and urethane, indicating that, at this dose, all bean milks were non-toxic.

The co-administration study was used to evaluate the mutagenic potential of each bean milk. Parental strains were mated on the regular medium, and then the F1 larvae were transferred and cultured on the experimental medium containing DI (negative control), urethane (positive control) and bean milk. Wings were collected and scored for mutant spots as small, large and twin spots. The data showed that total spots were 0.23 spots/wing in the DI group; total spots increased to 11.35 spots/wing in urethane-treated flies (Table 1). All types of mutant spots covering small, large and twin were observed. Urethane is a well-known mutagen and carcinogen; thus, our data confirmed the mutagenicity of urethane [29]. Flies exposed to bean milks showed low mutant spots compared to the DI control (Table 1), implying that all bean milks showed no mutagenicity.

### 3.2. Mutagenic Evaluation of Bean Milks Using the Pre-Feeding Study

The third instar larvae were used in the study; thus, there was a chance that the larvae were too old to eat the bean extract. These larvae underwent pupation without any exposure to bean milks. Therefore, to avoid false negative results from the co-administration study, a pre-feeding study was also used. In the pre-feeding study, the first instar larvae had more time to consume the medium compared with the co-administration study. Using this approach, the DI control exhibited total spots at 0.15 spots/wing; significantly increased total spots/wing was observed in the urethane-treated group (Table 2). All types of mutant spots were clearly presented in this group, suggesting that urethane behaved as a mutagen in both treatment types (Table 1 and Table 2). Flies treated with bean milks also showed low mutant spots compared to the DI control (Table 2) except for milks obtained from mung bean and red kidney bean. Interestingly, the number of small spots increased in flies that received mung bean (0.30 spots/wing), indicating some DNA damage in this group. However, statistical diagnoses of total spots in both mung bean and red kidney bean treatments were inconclusive.

### 3.3. Anti-Mutagenic Evaluation of Bean Extracts Using the Co-Administration Study

The same wing spot assay was used to explore the anti-mutagenicity of bean milks to support further food applications of beans using urethane as a model mutagen. The anti-mutagenicity of bean extracts was first evaluated using the co-administration study following the experimental procedure described in Figure 3. The F1 larvae obtained from the regular medium were subsequently fed with the experimental medium containing 20 mM urethane and each bean milk. Results in Table 3 show that urethane induced total spots at 9.11 spots/wing. Intriguingly, the mixture of urethane and bean milk reduced the number of mutant spots ranging from 6.25 to 8.00 spots/wing (Table 3). Milks derived from red kidney bean, mung bean and soybean showed high anti-mutagenicity against urethane by reducing the number of mutant spots to 24.57%, 23.99% and 27.75%, respectively, while the lowest anti-mutagenicity was observed in peanut milk. Results in Table 3 also show that small single spots visibly decreased when all bean milks were co-treated with urethane. 

### 3.4. Anti-Mutagenic Evaluation of Bean Extracts Using the Pre-Feeding Study

The pre-feeding study was used to reduce the possibility of differences in bean milk exposure. This approach divided the experiment into two types as pre-feeding study type I and type II. In the type I pre-feeding study, the parental strains were mated on the experimental medium containing bean milk, and the obtained third instar larvae were fed on the medium with urethane but without bean milks. In type II pre-feeding study, the parental strains were mated on the experimental medium containing bean milk, and then the obtained third instar larvae were continuously fed on the medium with urethane and each bean milk (Figure 4).

Results in Table 4 reveal that the overall anti-mutagenic properties against urethane of bean milks assayed by the type I pre-feeding study decreased compared to the co-administration study (Table 3). Highest anti-mutagenicity was recorded in peanut milk (19.08%); no anti-mutagenicity was observed in mung bean milk (−6.07%). The data suggested that each bean milk residual had a minor effect as an anti-mutagenic agent in vivo.

Interestingly, remarkable anti-mutagenicity toward urethane was detected using the type II pre-feeding study (Table 4). Anti-mutagenicity ranged between 6.87 and 76.59%. Mung bean milk again showed the lowest anti-mutagenic activity; soybean milk showed the highest (76.59%). Black and red kidney bean milks inhibited urethane-induced mutation by 43.51% and 46.06%, respectively.

### 3.5. Antioxidant Activities and Total Phenolic Contents of Bean Milks

The FRAP assay is an important test for assessing the scavenging capacity of free radicals and the metal ion excretion capacity of plant extracts [30]. FRAP is a stable free radical exhibiting a color with maximum absorption at 600 nm. This dark blue color typically fades when antioxidants quench FRAP free radicals by the single electron transfer reaction (SET). Data showed that the bean extracts displayed FRAP values ranging from 0.241 ± 0.015 to 0.029 ± 0.001 mM FeSO_4_ equivalent/g DW (Table 5). Red kidney bean showed the highest FRAP activity at 0.241 mM FeSO_4_ equivalent/g DW; soybean showed the lowest by approximately 8 times compared to red kidney bean.

Phenolic contents are always hypothesized to be key compounds in quenching free radicals [31]. TPCs of bean extracts were determined. Results in Table 5 show that all bean extracts contained phenolics, with the gallic acid calibration curve ranging from 6.20 to 103.73 mg GAE/g DW. Red kidney bean extract contained the highest amount of phenolics; soybean exhibited the lowest phenolic contents. Phenolics contain hydroxyl groups that are responsible for the antioxidant functions of plants. This correlation was also observed in red kidney bean and soybean (Table 5).

### 3.6. Principal Component Analysis (PCA)

**Figure 6:**
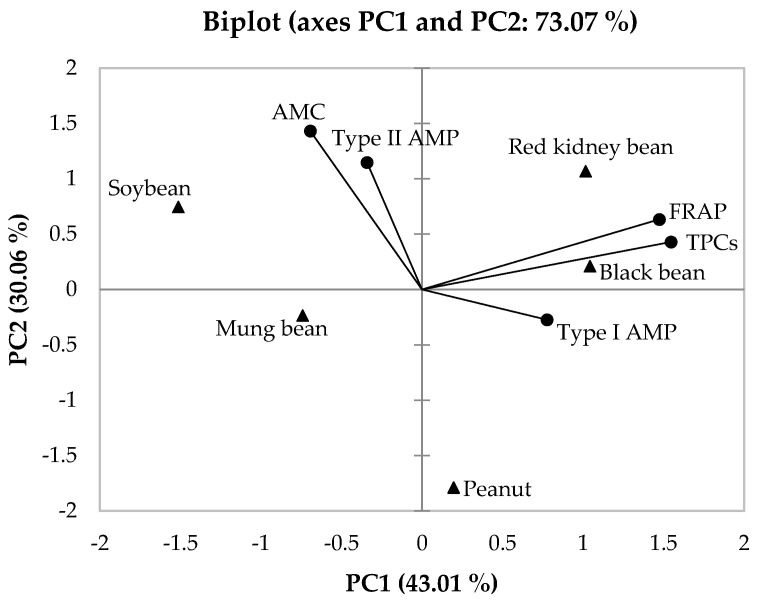
The biplot from principal component analysis (PCA) derived from mean values of all variables (% anti-mutagenicity, FRAP activities and TPCs) of five bean milks. AMC: % anti-mutagenicity from co-administration study; Type I AMP: % anti-mutagenicity from type I pre-feeding study; Type II AMP: % anti-mutagenicity from type II pre-feeding study; FRAP: ferric reducing antioxidant power; TPCs: total phenolic contents.

To draw conclusions from the data, mean values of percentage anti-mutagenicity, FRAP activities and TPCs were analyzed by PCA to determine correlations. Figure 6 shows the biplot derived from PCA with two axes (PC1 and PC2). The total combination between PC1 and PC2 was 73.07%, indicating a good representation of all analyzed data. Our data showed that TPCs and FRAP activities were closely located, indicating a positive correlation (Figure 6). Bean milks from black bean and red kidney beans were also located close to % anti-mutagenicity from type I pre-feeding study (Type I AMP), FRAP activities and TPCs, implying positive correlation between Type I AMP, FRAP activities, TPCs, black bean and red kidney bean. Thus, high TPCs and FRAP activities may contribute to the anti-mutagenicity observed in both black bean and red kidney bean. Conversely, soybean milk, which exhibited high anti-mutagenicity in all three strategies, was positioned far away from FRAP activities and TPCs, suggesting poor correlation between percentage anti-mutagenicity and FRAP activities and TPCs (Figure 6). Other phytochemicals in soybean may also play a significant role in anti-mutagenesis against urethane.

## 4. Discussion

DNA mutations and chromosomal aberration are the underlying causes of carcinogenesis. Thus, consumption of food with anti-mutagenic properties is a possible approach to mitigate cancer incidences. Beans are a popularly consumed plant-based food throughout the world because of their short harvesting time, good taste, high nutrient content and contained phytochemicals. However, the anti-mutagenic properties of beans are limited, and in vivo data are lacking. Hence, this study investigated the mutagenic, anti-mutagenic and antioxidant properties of five bean milks commonly consumed in Thailand, namely black bean (*Phaseolus vulgaris* L.), mung bean (*Vigna radiata* (L.) R. Wilczek), peanut (*Arachis hypogaea* L.), red kidney bean (*Phaseolus vulgaris* L.) and soybean (*Glycine max* (L.) Merr.). Several genotoxicity tests can be used to evaluate the mutagenicity and anti-mutagenicity of chemicals; however, in the present study, we employed the SMART or wing spot assay. Advantages of SMART over other genotoxicity tests include (i) several types of DNA mutation can be detected within SMART [19], (ii) inexpensive compared to other in vivo models, and (iii) the *Drosophila* with high bioactivation partially represents mammalian xenobiotic biotransformation. Five strains of bacteria are needed for mutagenicity testing using Ames test, which is an assay recommended by the Organization for Economic Co-operation and Development (OECD), to cover many types of mutations; comet assay or chromosomal aberration test are mainly detect only DNA breaks [19]. Moreover, SMART is a rapid and inexpensive in vivo assay compared to the mutagenic testing in rodents [32]. Additionally, there are two types of mutagens including direct-acting and indirect-acting mutagens. The fruit flies in standard cross can be used to detected only direct-acting mutagens; *Drosophila* strains with high bioactivation can detect both types of mutagens. Thus, according to the principles of the 3Rs (Replacement, Reduction and Refinement), *Drosophila* strains with high bioactivation, which can be used to detect both direct-acting and indirect-acting mutagens were used in this study [19]. Moreover, in the Ames test or hypoxanthine-guanine phosphoribosyltransferase (HPRT) gene mutation assay, the mouse liver homogenate is essentially needed to detect indirect-acting mutagens. Although there are numerous advantages of SMART, its main drawbacks are that it is labor- and time-intensive and only wing tissues were determined. Our results from SMART indicated that all bean milks were genome-safe. Milks derived from soybean, red kidney bean and black bean showed high anti-mutagenic activities against urethane; mung bean was devoid of this property.

Our results indicated that all bean milks were genome-safe because they did not induce DNA mutations in the *Drosophila* wing imaginal disc in both the co-administration and pre-feeding studies (Table 1 and Table 2). The larvae had more time to consume bean milk in the pre-feeding study compared to the co-administration study. In accordance with our data, an acetone extract of whole bean seed and seed coat of black bean, peanut, red kidney bean, soybean and mung bean also showed no DNA mutations in *Drosophila* with high bioactivation, both in the co-administration and pre-feeding study [33]. Acetone as a solvent has an advantage over hot water extract (in this study) because it can extract both polar and non-polar compounds; hot water mainly extracts polar compounds, supporting the findings (Table 1 and Table 2) that all tested beans were devoid of genotoxicity. Complete data on genotoxicity testing of all selected beans in higher animal models are unavailable but some studies confirmed our findings. Mice treated with 20% black bean cv. Ouro Negro exhibited no induction of micronucleated polychromatic erythrocytes (a biomarker for chromosomal breaks) isolated from bone marrow and DNA lesions in leukocytes [34]. Two cultivars of peanut (Manigran and Granoleico) were extracted with ethanol and subsequently treated in Balb/C mice at the highest dose of 2000 mg/kg. Results showed no increase of micronucleus induction in isolated bone marrow and no tail moment induction (assayed by comet assay) in blood samples of mice exposed to ethanolic peanut extract compared with the control [35]. In addition to animal model, soybean milk was also tested for their DNA-damaging effects in human in a pilot study. Subjects consumed soya milk 1 L/d for 4 weeks, then the lymphocytes were subjected for comet assay. The data showed that soya bean milk did not induce endogenous DNA strand breakage in healthy subjects [36]. Thus, taken together, the data suggested that all bean milks were genome-safe in vivo.

We further explored the anti-mutagenic properties of each bean milk against a model food mutagen, urethane, using three types of strategies, namely co-administration, type I pre-feeding and type II pre-feeding studies, as depicted in Figure 3 and Figure 4. Results revealed that soybean milk exhibited high inhibitory activities against urethane-induced DNA mutations in all these three strategies followed by black and red kidney beans (Table 3 and Table 4). Intriguingly, the anti-mutagenic properties of most bean milks, except mung bean were enhanced when type II pre-feeding was employed. In particular, soybean milk inhibited urethane-induced mutation at 76.59%, 27.75% and 11.56% in type II pre-feeding, co-administration and type I pre-feeding, respectively. The same pattern was also observed in black and red kidney beans. The data indicated that prolonged consumption of bean milk exhibited higher anti-mutagenic potential against urethane. Short or intermittent consumption may reduce anti-mutagenic properties of each bean milk, especially in mung bean (Table 3 and Table 4). To the best of our knowledge, this is the first report detailing the anti-mutagenic properties of bean milk against a food mutagen. To support our findings, soyasapogenol B, obtained from soybean by-products, displayed anti-mutagenic properties against the direct-acting mutagen 2-acetoxyacetylaminofluorene (2AAAF) in Chinese hamster lung cells [37]; fermented black bean (koji) extracted with methanol inhibited 4-nitroquinoline-*N*-oxide (4-NQO) or benzo[a]pyrene induced mutations (over 70% inhibition) in *Salmonella typhimurium* TA98 and TA100 with S9 liver homogenate [38]. Urethane, 2AAAF, 4-NQO and benzo[a]pyrene can bind to DNA, leading to the formation of bulky DNA adducts. Therefore, soybean could disrupt the formation of bulky DNA adducts. Further investigations on other types of DNA damage induced by food compounds, such as alkylated bases, require investigation to enhance the health benefits of soybean. The common bean (*Phaseolus vulgaris* L.) belongs to the same family as red kidney bean and black bean, and the seed coat inhibited aflatoxin B_1_-induced DNA mutations in *S. typhimurium* TA98 and TA100 with bioactivation system [39,40].

Anti-mutagenic compounds inhibit mutagenic agents through several mechanisms, namely (i) inactivation of mutagens, (ii) binding to mutagens before they induce DNA damage, and (iii) antioxidant potency [41]. Hence, we tested whether TPCs and antioxidant properties (FRAP values) of bean milks contributed to the obtained anti-mutagenic properties of each bean milk. Black bean and red kidney bean milks showed positive correlation between TPCs, FRAP value and percentage anti-mutagenicity (Figure 6), implying that the phenolic contents presented in black bean and red kidney bean may quench the genotoxicity of urethane via an antioxidant pathway. Interestingly, among the nine common bean varieties, black and red kidney beans possessed the highest amounts of phytochemicals such as total phenolics, total flavonoids and anthocyanins that contributed to their high antioxidant activity involving FRAP and 2,2-diphenyl-1-picrylhydrazyl (DPPH) scavenging activity [42]. High performance liquid chromatography-electrospray ionization mass spectrometry (HPLC-ESI/MS) with diode array detection (DAD) results demonstrated that the major phytochemicals in black beans were hydroxycinnamic acids and 3-*O*-glucosides of delphinidin, petunidin and malvidin; hydroxycinnamic acids, diglycosides of kaempferol and quercetin were found in dark red kidney beans [43]. By contrast, soybean milk exhibited low TPCs and FRAP activity but showed higher anti-mutagenicity than both black and red kidney beans, suggesting that phenolics and antioxidants might not act as bioactive agents to suppress urethane genotoxicity. Soybean is well-known for its isoflavone contents, namely daidzein, daidzin, malonyldaidzin, genistein, genistin, malonylgenistin, glycitein and glycitin. These might not be detected using spectrophotometric techniques, as in the present study, and require HPLC analysis [44,45]. Urethane is an indirect-acting mutagen or promutagen, which is activated by cytochrome P450, leading to the formation of l,*N*^6^-ethenoadenosine and 3,*N*^4^-ethenocytidine DNA adducts [46,47,48]. Thus, inhibition of cytochrome P450 could reduce the formation of urethane-induced DNA adducts. Genistein and daidzein have been reported to directly interact with and inhibit cytochrome P450 function in a noncompetitive manner [49]; genistein can suppress the expression of several cytochrome P450 enzymes in hepatic cells such as CYP1A1, CYP1B1, CYP2E1, CYP2D6 and CYP3A4 [50]. Hence, soybean milk may reduce urethane-induced DNA damage through inhibition of cytochrome P450 instead of antioxidant pathways such as black and red kidney beans.

Although we stated that all tested bean milks lacked mutagenicity, our findings cannot be extrapolated to the bean seeds as a whole since our samples were prepared by imitating home bean milk. As a result, the seed cakes were not tested. Further research using whole bean seed may be able to overcome this limitation. The present study provides the prompt idea that some bean milks made from soybean, red and black kidney bean could inhibit urethane-induced mutations. Urethane can be found in alcoholic beverages and fermented food products. It has been reported that pickled vegetables and salted fish consumption are linked to an increased risk of gastric cancer occurrence [51]. Thus, drinking soybean milk during or after fermented food consumption might prevent DNA mutation and possibly gastric cancer. Investigation into clinical research is beneficial.

## 5. Conclusions

Milks made from five bean varieties commonly consumed in Thailand, namely black bean, red kidney bean, mung bean, peanut and soybean, were assessed using *Drosophila* with high bioactivation and found to be genome-safe. High anti-mutagenic properties against a food-derived mutagen, urethane, were observed in milk produced from black bean, red kidney bean and soybean. Anti-mutagenicity shown by black bean and red kidney beans was associated with their TPCs and antioxidant activities; other compounds in soybean might also contribute to anti-mutagenicity against urethane. More thorough investigations on the anti-mutagenic effects of bean milks against other food mutagens are required to enhance the health benefits of beans and develop bean-based functional foods for mutation reduction.

## Figures and Tables

**Table 1:** Frequency of mutant spots observed in trans-heterozygous, high bioactivation (HB) flies treated with DI, urethane or bean milks in the co-administration study.

Samples	Number of Wings	Frequency of Mutant Spots per Individual (Number of Spots) ^#^			
		Small Single (1–2 Cells)	Large Single (>2 Cells)	Twin	Total Spots
DI (negative control)	40	0.13 (5)	0.10 (4)	0.00 (0)	0.23 (9)
Urethane	40	9.03 (361)+	1.30 (52)+	1.03 (41)+	11.35 (454)+
Black bean	40	0.15 (6)i	0.06 (2)i	0.00 (0)−	0.20 (8)i
Red kidney bean	40	0.03 (1)−	0.00 (0)−	0.00 (0)−	0.03 (1)−
Mung bean	40	0.13 (5)−	0.00 (0)−	0.03 (1)−	0.15 (6)i
Peanut	40	0.08 (3)−	0.03 (1)−	0.00 (0)−	0.10 (4)−
Soybean	40	0.10 (4)−	0.00 (0)−	0.00 (0)−	0.10 (4)−

^#^ Statistical diagnoses using estimation of spot frequencies and confidence limits comparing to DI (negative control); + = positive; − = negative; i = inconclusive. Probability levels: α = β = 0.05. One-sided statistical test “m” is an increased mutation frequency compared with the spontaneous frequency (m times). The data were collected from two independent experiments (*n* = 2).

**Table 2:** Frequency of mutant spots observed in trans-heterozygous, high bioactivation (HB) flies treated with DI, urethane or bean milks in the pre-feeding study.

Samples	Number of Wings	Frequency of Mutant Spots per Individual (Number of Spots/Wing) ^#^			
		Small Single (1–2 Cells)	Large Single (>2 Cells)	Twin	Total Spots
DI (negative control)	40	0.08 (3)	0.08 (3)	0.00 (0)	0.15 (6)
Urethane	40	11.05 (442)+	2.13 (85)+	1.58 (63)+	14.75 (590)+
Black bean	40	0.18 (7)i	0.08 (3)−	0.00 (0)−	0.25 (10)i
Red kidney bean	40	0.08 (3)−	0.00 (0)−	0.03 (1)−	0.08 (3)−
Mung bean	40	0.30 (10)+	0.05 (2)−	0.00 (0)−	0.30 (12)i
Peanut	40	0.13 (5)i	0.03 (1)−	0.00 (0)−	0.15 (6)−
Soybean	40	0.10 (4)−	0.00 (0)−	0.03 (1)−	0.10 (4)−

^#^ Statistical diagnoses using estimation of spot frequencies and confidence limits comparing to DI (negative control); + = positive; − = negative; i = inconclusive. Probability levels: α = β = 0.05. One-sided statistical test “m” is an increased mutation frequency compared with the spontaneous frequency (m times). The data were collected from two independent experiments (*n* = 2).

**Table 3:** Frequency of mutant spots observed in trans-heterozygous, high bioactivation (HB) flies treated with urethane alone or bean milk together with urethane in the co-administration study.

Samples	Number of Wings	Frequency of Mutant Spots per Individual (Number of Spots/Wing)				Anti-Mutagenicity (%)
		Small Single (1–2 Cells)	Large Single (>2 Cells)	Twin	Total Spots	
Urethane	38	5.421 (206)	2.789 (106)	0.894 (34)	9.110 (346)	
Black bean	39	4.589 (179)	1.897 (74)	0.846 (33)	7.333 (286)	17.34
Red kidney bean	40	3.325 (133)	2.425 (97)	0.775 (31)	6.525 (261)	24.57
Mung bean	40	2.500 (100)	2.825 (113)	1.250 (50)	6.575 (263)	23.99
Peanut	40	4.275 (171)	2.425 (97)	1.300 (52)	8.000 (320)	7.51
Soybean	40	3.500 (140)	1.850 (74)	0.900 (36)	6.250 (250)	27.75

The data were collected from two independent experiments (*n* = 2).

**Table 4:** Frequency of mutant spots observed in trans-heterozygous, high bioactivation (HB) flies following the type I and type II pre-feeding studies.

Samples	Number of Wings	Frequency of Mutant Spots per Individual (Number of Spots/Wing)				Anti-Mutagenicity (%)
		Small Single (1–2 Cells)	Large Single (>2 Cells)	Twin	Total Spots	
**Type I**						
Urethane (positive control)	37	6.189 (229)	2.108 (78)	1.054 (39)	9.351 (346)	
Black bean	37	5.756 (213)	1.324 (49)	0.648 (24)	7.730 (286)	17.34
Red kidney bean	40	5.080 (203)	1.430 (57)	1.100 (44)	7.600 (304)	12.14
Mung bean	40	6.450 (258)	1.330 (53)	1.400 (56)	9.180 (367)	−6.07
Peanut	40	5.375 (215)	1.100 (44)	0.525 (21)	7.000 (280)	19.08
Soybean	40	6.250 (250)	1.850 (74)	0.675 (27)	7.650 (306)	11.56
**Type II**						
Urethane (positive control)	40	6.150 (246)	2.200 (88)	1.480 (59)	9.830 (393)	
Black bean	40	3.730 (149)	1.280 (51)	0.550 (22)	6.050 (222)	43.51
Red kidney bean	40	4.130 (165)	0.800 (32)	0.380 (15)	5.300 (212)	46.06
Mung bean	40	5.975 (239)	2.300 (92)	0.875 (35)	9.150 (366)	6.87
Peanut	40	5.450 (218)	1.925 (77)	0.975 (39)	8.350 (334)	15.01
Soybean	40	1.800 (72)	0.350 (14)	0.150 (6)	2.300 (92)	76.59

The data were collected from two independent experiments (*n* = 2).

**Table 5:** Ferric reducing antioxidant power (FRAP) values and total phenolic contents (TPCs) of each bean milk.

Samples	FRAP Values (mM FeSO_4_ Equivalent/g DW)	Total Phenolic Contents (mg GAE/g DW)
Black bean	0.207 ± 0.006 ^B^	93.12 ± 0.25 ^B^
Red kidney bean	0.241 ± 0.015 ^A^	103.73 ± 2.27 ^A^
Mung bean	0.100 ± 0.004 ^C^	43.69 ± 0.72 ^C^
Peanut	0.081 ± 0.007 ^D^	43.45 ± 3.56 ^C^
Soybean	0.029 ± 0.001 ^E^	6.20 ± 1.43 ^D^

The data were collected from three independent experiments and are expressed as mean ± SD (*n* = 3). Capital letters denote values that are significantly different from each other at *p* < 0.05 calculated by one-way analysis of variance (ANOVA) and Duncan’s multiple comparison test. DW: dry weight; GAE: gallic acid equivalent.

## Data Availability

Data are contained within this article.
